# Professional future of military personnel suffering from post-traumatic stress disorder

**DOI:** 10.1192/j.eurpsy.2025.1956

**Published:** 2025-08-26

**Authors:** Z. Fourati, S. Kamoun, H. Ziedi, M. Ahmed Ali, N. Khaterchi, A. Amri

**Affiliations:** 1 Occupational health department, Military Occupational Health Center, Tunis, Tunisia

## Abstract

**Introduction:**

Post-traumatic stress disorder (PTSD) is a disabling condition that develops after exposure to a traumatic event. Military personnel are particularly affected by this psychiatric condition, which profoundly alters their personal, social and professional lives. Professional redeployment is most often the decision taken to keep these military personnel in the army.

**Objectives:**

To identify the professional future of military personnel suffering from PTSD

**Methods:**

Retrospective descriptive study conducted at the Military Centre for Occupational Medicine and Occupational Safety between 2017 and 2022 among active military personnel requesting occupational redeployment.

**Results:**

We collected 22 cases of professional redeployment for PTSD, representing 24% of all requests for redeployment. Our study population was exclusively male, with a mean age of 30±9 years, mainly from the army (19 cases), divided into officers (3 cases), non-commissioned officers (12 cases) and enlisted men (7 cases). The traumatic event responsible for the PTSD was a mine explosion (8 cases), a road accident (6 cases), a gunshot wound (6 cases), a fall from a parachute (1 case) and an air accident (1 case). All patients had previously been exempted from certain military duties, mainly carrying weapons (22 cases), guards duty (5 cases) and driving vehicles (5 cases). When the decision to reclassify was taken, the military personnel was affected to a mainly administrative post (19 cases), a gardening post (1 case), a plumbing post (1 case) and a catering post (1 case).

**Conclusions:**

This study highlights the fact that PTSD in the military represents a serious and complex challenge that requires special attention. It is imperative to put in place preventive measures and provide appropriate management of PTSD. That can support the military personnel affected and maintain the operational capability of the troops.

**Disclosure of Interest:**

None Declared

